# Targeting PON2 with Vutiglabridin Restores Mitochondrial Integrity and Attenuates Oxidative Stress-Induced Senescence

**DOI:** 10.3390/antiox14111288

**Published:** 2025-10-27

**Authors:** Jin-Woong Heo, Hyeong Hwan Kim, Jae Ho Lee, Hyeong Min Lee, Hyung Soon Park, Chang-Hoon Nam

**Affiliations:** 1 Aging and Immunity Laboratory, Department of New Biology, Daegu Gyeongbuk Institute of Science and Technology, Daegu 42988, Republic of Korea; hjw001107@dgist.ac.kr (J.-W.H.); khh11171@dgist.ac.kr (H.H.K.); 2 Research Department, Glaceum Incorporation, Suwon 16675, Republic of Korea; jaeholee@glaceum.com (J.H.L.); lohmjy325@glaceum.com (H.M.L.); hspark@glaceum.com (H.S.P.); 3 New Biology Research Center, Daegu Gyeongbuk Institute of Science and Technology, Daegu 42988, Republic of Korea

**Keywords:** cellular senescence, reactive oxygen species, mitochondrial dysfunction, paraoxonase 2, vutiglabridin

## Abstract

Oxidative stress-induced mitochondrial dysfunction has been identified as a central driver of cellular senescence and age-related degeneration. The present study investigated the potential of vutiglabridin, a paraoxonase 2 (PON2) agonist, to mitigate reactive oxygen species (ROS)-induced senescence in human LO2 hepatocytes. The process of senescence was induced by the administration of hydrogen peroxide, followed by the recovery of the cells in fresh medium. The levels of intracellular ROS, the senescence-associated β-galactosidase staining, the p16/p21 expression, and the mitochondrial morphology were the focus of a comprehensive assessment utilizing a range of analytical techniques, including microscopy, quantitative PCR, and Western blotting. The present study demonstrated that the administration of vutiglabridin resulted in a dose-dependent reduction in attenuation of the expression of senescence markers. Transmission electron microscopy (TEM) and stimulated emission depletion (STED) imaging revealed the preservation of mitochondrial structure and network connectivity in cells treated with vutiglabridin. These effects were absent in PON2 knockout cells, confirming that vutiglabridin’s action requires functional PON2. The present study demonstrates that vutiglabridin alleviates oxidative stress-induced cellular senescence by preserving mitochondrial integrity and redox balance via a PON2-dependent mechanism. This study lends further support to the investigation of the PON2 pathway as a therapeutic target in age-related cellular dysfunction.

## 1. Introduction

Cellular senescence is defined as a state of stable cell cycle arrest in which cells cease to proliferate but remain metabolically active. This process can be triggered by a variety of intrinsic and extrinsic stimuli, including telomere attrition, DNA damage, inflammation, and oxidative stress [1]. Experimentally, the extent of senescence can be quantified by assessing senescence-associated β-galactosidase (SA-β-gal) activity, analyzing the expression of cyclin-dependent kinase inhibitors (CDKIs) such as p16 and p21, detecting DNA damage markers like γ-H2AX, and observing characteristic morphological changes [2].

While cellular senescence plays a critical role in maintaining tissue homeostasis, the chronic accumulation of senescent cells acts as a key contributor to chronic inflammation, functional decline of tissues, and the progression of various age-related diseases [3]. Senescent cells secrete a broad range of cytokines, chemokines, and matrix-degrading enzymes collectively known as the senescence-associated secretory phenotype (SASP), which can impair the function of neighboring cells and disrupt the tissue microenvironment [4]. Therefore, therapeutic strategies aimed at alleviating senescence are essential for the prevention and treatment of aging-associated disorders, and considerable research is currently focused on the identification and development of senescence-modulating compounds.

One of the key molecular mechanisms underlying cellular senescence is the accumulation of ROS generated during metabolic processes [5]. Mitochondria-derived ROS are essential for normal cellular signaling and maintenance of homeostasis; however, excessive ROS accumulation can cause damage to DNA, proteins, and lipids, ultimately impairing cellular function. In senescent cells, the mitochondrial electron transport chain operates less efficiently, leading to increased electron leakage and overproduction of ROS such as superoxide. Additionally, aging is associated with a decline in the expression of antioxidant enzymes, including superoxide dismutase and catalase, which weakens the cell’s capacity to eliminate ROS [6].

This accumulation of ROS activates inflammatory signaling pathways, such as NF-κB, which in turn promotes the secretion of SASP factors and further amplifies ROS production in a feed-forward manner [7]. Notably, ROS can activate the ATM/ATR pathways, triggering the DNA damage response (DDR), which leads to the upregulation of p53 and p21, thereby promoting cellular senescence [7]. Thus, maintaining redox homeostasis is considered a critical strategy for delaying the onset of aging.

Mitochondria experience notable structural and functional changes as part of the aging process. A decrease in mitochondrial membrane potential, diminished efficiency of the electron transport chain, and compromised mitochondrial quality control mechanisms all lead to excessive production of ROS and a weakened antioxidant defense system [6]. Typically, 1% to 5% of total oxygen is converted into ROS under normal physiological conditions [8]. However, when mitochondrial function is compromised for various reasons, this percentage rises, leading to increased ROS production and accumulation. The buildup of ROS causes mitochondrial DNA mutations, damage to the electron transport chain, alterations in mitochondrial membrane permeability and structure, and disruption of calcium homeostasis [8]. These consequences collectively result in a decline in mitochondrial function. Consequently, in environments characterized by high levels of ROS, such as those found in cellular senescence, maintaining mitochondrial function and preserving mitochondrial integrity and morphology can mitigate ROS production and prevent its accumulation.

The interplay between ROS and mitochondrial dysfunction forms a critical feedback loop that drives and sustains cellular senescence. Mitochondrial dysfunction not only results in increased ROS production, but ROS in turn further impairs mitochondrial components, reinforcing senescent phenotypes. This self-amplifying cycle is recognized as a hallmark of the senescence-associated mitochondrial dysfunction (SAMD) pathway [9]. Senescent cells often exhibit enlarged and dysmorphic mitochondria, indicative of impaired mitophagy and defective mitochondrial turnover, both of which exacerbate oxidative stress [10]. In particular, accumulation of ROS activates DDR pathways and inflammatory signaling cascades, such as NF-κB, thereby promoting the SASP [11]. Furthermore, mitochondrial-derived ROS have been shown to act as upstream effectors in the activation of p53/p21 and p16INK4a pathways, linking redox imbalance to irreversible growth arrest [12]. This connection between mitochondrial health and senescence emphasizes the importance of targeting the link between ROS and mitochondria in aging-related therapeutic strategies. Therefore, interventions that can restore mitochondrial integrity and reduce oxidative stress hold significant potential to delay or reverse cellular senescence.

PON2, a member of the paraoxonase family, is noted for its lactonase and redox activities [13]. Research has shown that PON2 serves a protective function in mitochondria, particularly concerning the generation of ROS and the process of apoptosis [14]. PON2 is crucial for regulating mitochondrial function and sustaining mitochondrial homeostasis. Depletion or genetic knockout of PON2 leads to elevated mitochondrial ROS levels, impaired mitochondrial respiration, and reduced membrane potential, ultimately disrupting cellular metabolic balance [15]. Moreover, PON2 deficiency has been associated with decreased antioxidant defense capacity, contributing to oxidative stress accumulation and mitochondrial damage [16]. Recent studies have shown that PON2 localizes to the inner mitochondrial membrane and modulates coenzyme Q-dependent redox cycling, thereby directly influencing mitochondrial function [17]. In various cell types, including hepatocytes and retinal pigment epithelial (RPE) cells, loss of PON2 compromises mitophagy and autophagic flux, leading to the accumulation of dysfunctional mitochondria, impaired mitochondrial respiration, increased inflammation, and tissue-specific pathologies such as hepatic fibrosis and retinal dysfunction [18]. In human hepatocytes, PON2 modulates autophagy-related pathways, and its absence has been linked to both mitochondrial and metabolic dysfunction [19]. Similarly, in RPE cells, PON2 deficiency disrupts mitochondrial homeostasis and antioxidant defense mechanisms, contributing to progressive retinal degeneration [18]. These findings highlight the essential role of PON2 in preserving mitochondrial integrity under oxidative stress and support its potential as a therapeutic target for age-related mitochondrial decline and cellular senescence.

Vutiglabridin is an innovative anti-obesity agent developed through structural modification of glabridin [20], and recent findings have extended its therapeutic potential beyond metabolic disorders. As a PON2 agonist, vutiglabridin enhances mitochondrial function and contributes to cellular redox balance. In aged mice, vutiglabridin has been shown to alleviate age-related metabolic dysfunctions by improving mitochondrial complex I assembly, increasing NAD^+^ levels, and enhancing fatty acid metabolism, ultimately reducing systemic inflammation and oxidative stress [21]. In human dermal fibroblasts, long-term treatment with vutiglabridin mitigated cellular senescence by reducing p16 and p21 expression, restoring mitochondrial structure and function, and decreasing markers of oxidative damage. Furthermore, vutiglabridin improved circadian rhythm amplitude and normalized the period of basic helix–loop–helix ARNT like 1 (BMAL1) expression, suggesting a regulatory role in cellular timekeeping mechanisms closely linked to aging [22]. These results collectively suggest that vutiglabridin exerts anti-aging effects through the restoration of mitochondrial homeostasis, metabolic regulation, and circadian rhythm synchronization, positioning it as a promising therapeutic candidate for aging-related conditions. The objective of this study is to evaluate the hypothesis that the enhancement of PON2 function by vutiglabridin can restore mitochondrial activity and metabolic balance in senescent cells. By providing the first evidence that vutiglabridin can slow down the progression of cellular senescence and mitigate mitochondrial dysfunction along with various senescent characteristics, our results suggest a new approach centered on PON2 activation.

## 2. Materials and Methods

### 2.1. Cell Culture

LO2 cells (an immortalized normal liver cell line) were provided from Dr. Gu-Choul Shin (The Catholic University of Korea).

Cells were grown in Dulbecco’s modified Eagle medium (Welgene, Gyeongsan, Korea) supplemented with 10% fetal bovine serum (Gibco) and antibiotics (Welgene, Gyeongsan, Republic of Korea) at 37 °C, 5% CO_2_.

### 2.2. SA-β-Gal Assay

The SA-β-gal assay was performed with a senescence beta-galactosidase staining kit (Cell Signaling Technology, Danvers, MA, USA). β-galactosidase staining solution and the fixative solution were arranged according to the manufacturer’s instructions. After treating 1 mL of 1× fixative solution in cells seeded in a 6-well plate, they were washed twice with DPBS. Next, cells were incubated in a 37 °C dry incubator with 1 mL of X-gal staining solution. Quantification of SA-β-gal-positive cells was performed from three fields obtained from three independent experiments per sample, with at least 200 total cells analyzed under a bright-field microscope [23]. The number of SA-β-gal-positive cells was determined by precisely counting them using ImageJ software (version 1.54g, National Institutes of Health, Bethesda, MD, USA) after adjusting the color threshold to identify the blue-stained cells.

### 2.3. RNA Extraction and cDNA Synthesis

Total RNA extraction from cells was performed with an RNeasy mini kit (Qiagen, Hilden, Germany). The extracted total RNA was reverse-transcribed with TOPscriptTM Reverse Transcriptase Kit (Enzynomics, Daejeon, Republic of Korea) and Oligo dT primers.

### 2.4. Quantitative Real-Time PCR

Quantitative PCR was performed with TOPrealTM qPCR 2X premix (Enzynomics, Daejeon, Republic of Korea) and a CFX96 Touch Deep Well Real-Time PCR Detection System (Bio-Rad).

The following condition was used: 95 °C for 15 min, 44 cycles at 95 °C for 10 s, 60 °C for 15 s, and 72 °C for 30 s.

The primer sequence was as follows:

P21 (CDKN1A) forward: 5′-CCGCCCCCTCCTCTAGCTGT-3′

P21 (CDKN1A) reverse: 5′-CCCCCATCATATACCCCTAACACA-3′

Accession number: AF497972.1

P16 (CDKN2A) forward: 5′-CCCCGATTGAAAGAACCAGAGA-3′

P16 (CDKN2A) reverse: 5′-ACGGTAGTGGGGGAAGGCATAT-3′

Accession number: AB060808.1

GAPDH1 forward: 5′-GAAGGTGAAGGTCGGAGT-3′

GAPDH1 reverse: 5′-GAAGATGGTGATGGGATTTC-3′

Accession number: NM_002046

PON2 (Paraoxonase 2) forward: 5′-CGTTGTCTATGAAAGTGCTGA-3′

PON2 (Paraoxonase 2) reverse: 5′-GGTCTAAAATTCCCAGGACTCC-3′

Accession number: NM_000305.3

### 2.5. Confocal Microscopy and Electron Microscopy

Cells in each group were grown in 35 mm glass-bottomed culture dishes (NEST Biotechnology Co., Wuxi, China, 801001) to 50–60% confluency. The next day, cells were incubated with 200 nM MitoTracker (M7514; Invitrogen, Carlsbad, CA, USA) for 20 min, then washed with PBS, and then examined under a confocal microscope (Olympus Corporation, Tokyo, Japan) to investigate structural changes of mitochondria.

Ultrastructural analysis of mitochondria was carried out by transmission electron microscopy (TEM). The cells on the coverslip were fixed with 2.5% glutaraldehyde-mixed 2% paraformaldehyde solution for 1 h, followed by post-fixation in 2% osmium tetroxide for 1 h at 4 °C. The fixed cells were dehydrated with a graded ethanol series and then embedded into an epoxy medium (EMS, Hatfield, PA, USA). Embedded samples were sectioned (60 nm) with an ultra-microtome (Leica Microsystems, Wetzlar, Germany), and the sections were then viewed on a Tecnai 20 TEM (Thermo Fisher Scientific, Waltham, MA, USA) at 120 kV. Then they were double-stained with UranyLess (EMS, 22409) for 2 min and 3% lead citrate (EMS, 22410) for 1 min. Images were captured with a US1000X-P camera 200 (Gatan, Pleasanton, CA, USA).

### 2.6. Western Blot

Resuspending cultured cell pellets were prepared for protein lysates with SDS sample buffer (50 mM Tris-HCl 2% SDS, 0.1% bromophenol blue, 10% glycerol). Protein quantification was performed with an RC DC protein assay kit (Bio-Rad, Hercules, CA, USA). A total of 15 μg of protein in each sample was separated in 10% gradient Tris-glycine gel and transferred to a nitrocellulose membrane. Membranes were blocked with TBST buffer (20 mM Tris, 150 mM NaCl, 0.5% Tween-20) supplemented with 5% BSA and incubated with primary antibody. After washing with TBST, membranes were incubated with HRP-conjugated secondary antibodies. Similarly, after washing with TBST, an enhanced chemiluminescence solution (Thermo Scientific, Waltham, MA, USA) was added for detection. The primary antibodies are: anti-CDKN2A/p16INK4a antibody (EPR1473; abcam, Cambridge, UK, 1:1000 dilution), anti-PON2 antibody (EPR15295-82; abcam, Cambridge, UK, 1:1000 dilution), rabbit GAPDH (2118; Cell Signaling Technology, Danvers, MA, USA, 1:1000 dilution). The secondary antibodies are: peroxidase AffiniPure donkey anti-rabbit IgG (711-035-152; Jackson Immunoresearch, West Grove, PA, USA, 1:5000 dilution).

### 2.7. Cell Viability Assay

The cell viability with chemicals were evaluated by a 3-(4,5-dimethyl-2-thiazolyl)-2,5-diphenyl-2H-tetrazolium bromide (MTT) assay. LO2 (5000/well) cells were seeded in 96-well plates overnight. To detect the effects of peroxide (H1009; Sigma-Aldrich, St. Louis, MO, USA) on viability, cells were first co-cultured with different concentrations (4000, 2000, 1000, 800, 600, 400, 200, 100, 50, 0 μM) of peroxide (H1009; Sigma-Aldrich, St. Louis, MO, USA) for 24 h. After that, 100 μM of 3-(4,5-dimethyl-2-thiazolyl)-2,5-diphenyl-2H-tetrazolium bromide (M2128; Sigma-Aldrich, St. Louis, MO, USA) solution with DMEM media was added to the cells. After incubation for 4 h, the absorbance was observed at 562 nm with a microplate reader. Relative cell viability was calculated by this formula: experimental group absorbance at 562 nm/control group absorbance at 562 nm × 100%.

### 2.8. Senescence Induction in LO2 Cells

Senescence induction in LO2s were induced with peroxide (H1009; Sigma-Aldrich, St. Louis, MO, USA). To find the optimal concentration of peroxide, a cell viability assay with LO2s with peroxide was performed. The optimal peroxide concentration was determined as that which did not affect the viability of cells. After then, cells were treated with peroxide for 24 h and the medium was changed to fresh DMEM media to develop the senescence phenotypes. The procedure for inducing senescence with hydrogen peroxide is as follows: After seeding the cell, 600 µM hydrogen peroxide was treated with culture media for 24 h. The medium was then changed to one that did not contain hydrogen peroxide. LO2s were cultured and tested after 3, 5, and 7 days and defined as the 3-day group, 5-day group, and 7-day group, respectively.

### 2.9. Generation of PON2 Knockout (KO) LO2 Cell Line

PON2 knockout LO2 cell lines were generated using the PON2 double nickase plasmid (sc-403181-NIC; Santa Cruz Biotechnology, Dallas, TX, USA) and TurboFect™ transfection reagent (R0531; Thermo Fisher Scientific, Waltham, MA, USA). LO2 cells were seeded in 6-well plates and transfected with the plasmid complexed with TurboFect according to the manufacturer’s protocol. Following transfection, cells were cultured in medium containing puromycin (P8833; Sigma-Aldrich, St. Louis, MO, USA) at a concentration determined to selectively eliminate untransfected cells. After selection, surviving cells were individually seeded into 96-well plates at a density of one cell per well to allow for clonal expansion. Colonies derived from single cells were harvested and screened for PON2 knockout using Western blotting with a PON2-specific antibody and quantitative PCR using primers targeting PON2 transcripts. Clones confirmed to lack PON2 expression at both the protein and mRNA levels were selected for further experiments.

### 2.10. Statistical Analysis

All statistical analyses were performed using GraphPad Prism version 5.0 (GraphPad Software, San Diego, CA, USA). For comparisons between two groups, the two tailed Student’s *t*-test was used. Data are presented as the mean ± standard error (SE), and a *p*-value < 0.05 was considered statistically significant.

## 3. Results

### 3.1. Generation and Validation of PON2 Knockout Cells

**Figure 1:**
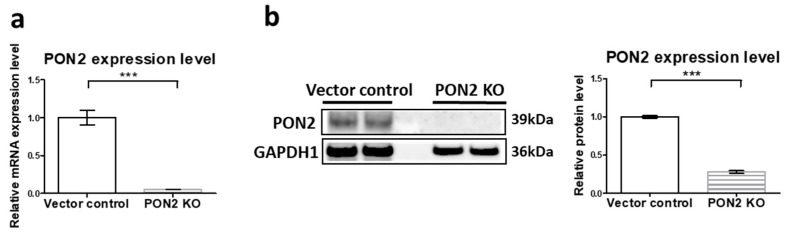
Validation of PON2 knockout in LO2 cells: (**a**) Relative mRNA levels of PON2 in vector control and PON2 KO LO2 cells measured by quantitative real-time PCR. Values in vector control were normalized to 1. Data are shown as the mean ± SE (n = 3, *** *p* < 0.001) by Student’s two-tailed *t*-test. (**b**) Representative Western blot showing PON2 protein expression in vector control and PON2 KO LO2 cells with GAPDH1 as the loading control. Right panel shows quantification of relative protein levels. Values in vector control were normalized to 1. Data are shown as the mean ± SE from three independent experiments (n = 3, *** *p* < 0.001) by Student’s two-tailed *t*-test.

PON2 is known to play a crucial role in maintaining cellular redox homeostasis and mitigating oxidative stress [24,25,26]. To investigate whether vutiglabridin’s anti-senescent effects are mediated through PON2 activation, we first established a PON2 KO model in LO2 hepatocytes using CRISPR/Cas9 technology. PON2 deletion was confirmed by a Western blot and qRT-PCR analysis (Figure 1). PON2 KO cells exhibited an over 80% reduction in PON2 protein expression compared with vector control cells (*** *p* < 0.001).

### 3.2. Treatment of Low-Concentration Hydrogen Peroxide Provokes ROS-Induced Senescence in LO2 Hepatocytes

**Figure 2:**
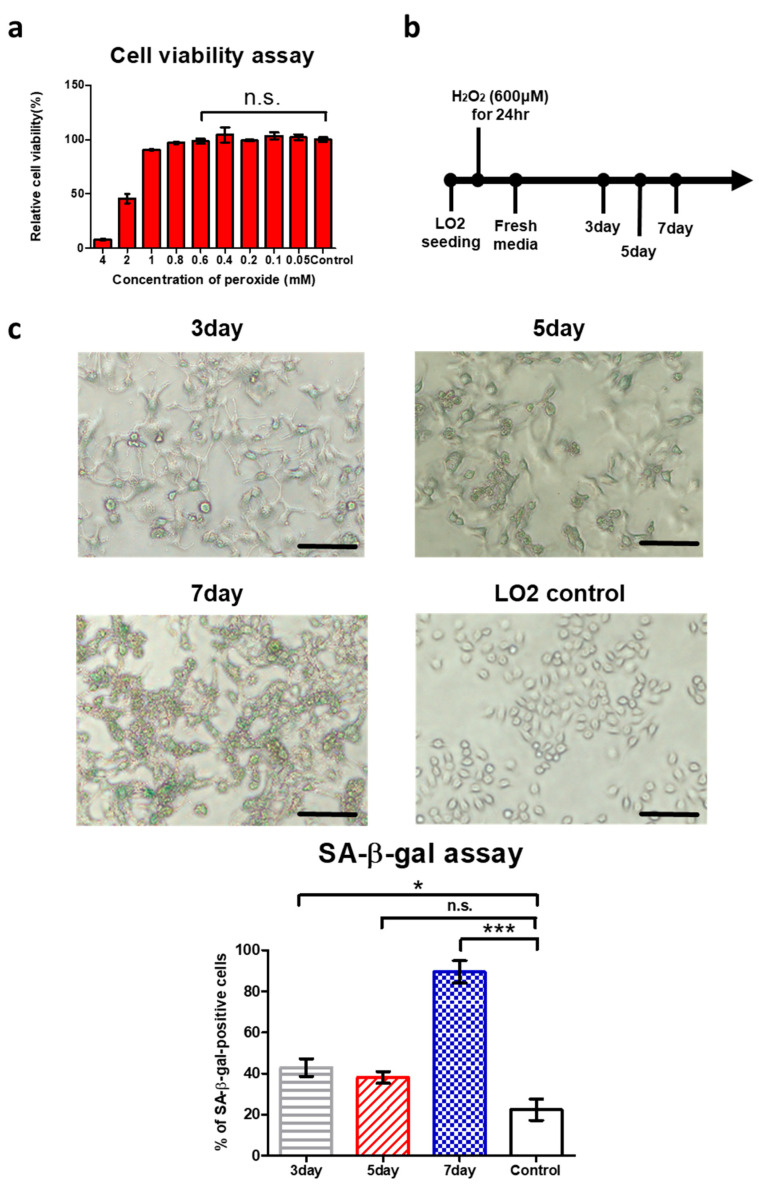

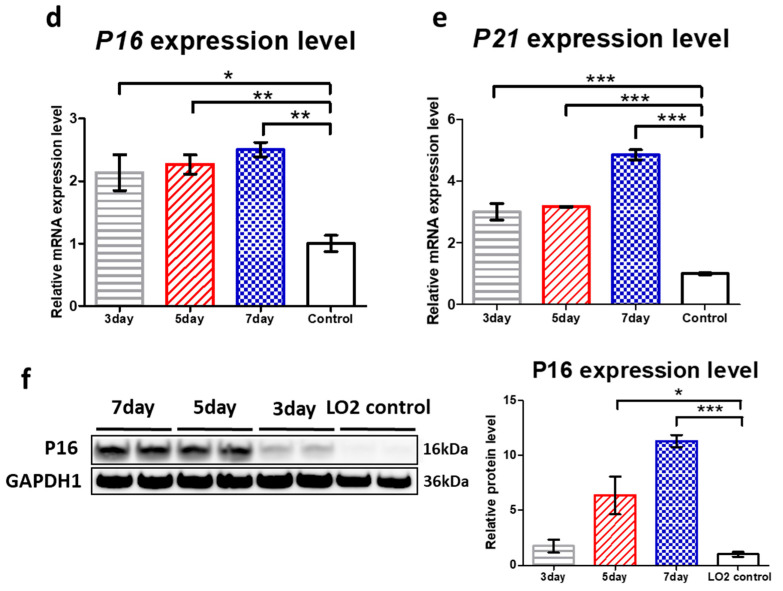
Hydrogen peroxide treatment in LO2 hepatocytes showed ROS-induced senescence. (**a**) LO2 cell viability with peroxide treatment was measured with an MTT assay. Relative cell viability was defined as experimental group/control group × 100%. Data are shown as the mean ± SE (n = 4, n.s. no significance). (**b**) Experimental scheme: LO2 cells were treated with 600 μM H_2_O_2_ for 24 h, followed by incubation in fresh media for 3, 5, or 7 days. (**c**) Photomicrograph of SA-β-gal staining of LO2s for 3, 5, 7 days with fresh media after exposing 600 μM hydrogen peroxide for 24 h. The scale bar represents 100 μm. Quantification of SA-β-gal-positive cells was performed from 3 representative fields, with at least 200 total cells analyzed. Results are expressed as the percentage of stained cells. Data are presented as the mean ± SE from three images. (n = 3, * *p* < 0.05, *** *p* < 0.001, n.s. no significance, by Student’s two-tailed *t*-test). (**d**,**e**) The relative gene expression of P16 and P21 in LO2s in the 3-day, 5-day, and 7-day groups and LO2 control groups. The comparison with the expression level in LO2 control group is adjusted to 1. Data are shown as the mean ± SE from three independent experiments (n  =  3, * *p* < 0.05, ** *p* < 0.01, *** *p* < 0.001, by Student’s two-tailed *t*-test). (**f**) Total lysates from 3-day, 5-day, and 7-day LO2 groups and LO2 control groups were subjected to Western blotting using specific antibodies for P16 and GAPDH1. Protein expression levels obtained from Western blotting were quantified. Values in the LO2 control were normalized to 1. Data are shown as the mean ± SE from four independent experiments. (n = 4, * *p* < 0.05, *** *p* < 0.001, by Student’s two-tailed *t*-test).

ROS induce damage to proteins, epigenetic machinery, nucleic acids, lipids, and proteins and accelerate cellular senescence and cell death [27]. Low-concentration, long-term treatment with hydrogen peroxide has been used as a representative method to induce cellular senescence by increasing the concentration of ROS in cells [28]. In fibroblasts treated with hydrogen peroxide for a long time, the expression of P16 and P21 increased, and the proportion of SA-β-gal-positive cells increased. This phenotype is similar to replicative senescence [29]. To induce ROS-induced senescence in LO2s, the concentration of hydrogen peroxide should be determined. Hydrogen peroxide that does not affect cell viability is a suitable concentration to induce senescence. To obtain the profit condition of hydrogen peroxide treatment for LO2s, the cell viability assay was performed. An amount of 0.05~0.6 mM hydrogen peroxide with culture media for 24 h did not affect the viability of LO2s (Figure 2a). Based on this result, 600 μM hydrogen peroxide was selected as the profit concentration. LO2 cells were treated with H_2_O_2_, and the assay was performed on days 3, 5, and 7, which were defined as the 3-day group, 5-day group, and 7-day group, respectively. (Figure 2b). Representative pictures of the 3, 5, and 7 groups and LO2 control groups are shown in Figure 2c. SA-β-gal-positive cells were increased during culture with fresh media (Figure 2c). The gene expression of P16 (CDKN2A) and P21 (CDKN1A) was measured in each of the 3-day, 5-day and 7-day fresh media groups. LO2s cultured with hydrogen peroxide showed higher P16 and P21 gene expression levels than the control group. With longer culture time, the P16 and P21 mRNA expression level of each group became higher (Figure 2d). Figure 2d shows fold changes in P16 mRNA levels of 2.13 (3-day group), 2.27 (5-day group), and 2.50 (7-day group) relative to the LO2 control, respectively. Figure 2e shows fold changes in P21 mRNA levels of 2.98 (3-day group), 3.17 (5-day group), and 4.85 (7-day group) relative to the LO2 control, respectively. The 5-day group showed significantly higher P16 and P21 mRNA levels than the LO2 control (Figure 2d, ** *p* < 0.01, *** *p* < 0.001). P16 protein levels were observed by Western blotting. Hydrogen peroxide-treated groups showed higher protein levels than the LO2 control (Figure 2f). Figure 2f shows fold changes in protein levels of 1.75 (3-day group), 6.36 (5-day group), and 11.30 (7-day group) relative to the LO2 control, respectively. The 5-day group showed significantly higher levels of P16 compared with the LO2 control (Figure 2f, ** *p* < 0.01). The increasing ratio of SA-β-gal-positive cells and the higher relative mRNA expression, protein level of P16, and genes in the hydrogen peroxide-treated LO2 group show that treatment with optimal concentration of hydrogen peroxide induces ROS-induced senescence in LO2 cells.

### 3.3. Vutiglabridin Attenuates H_2_O_2_-Induced Cellular Senescence in LO2 Cells via a PON2-Dependent Mechanism

**Figure 3:**
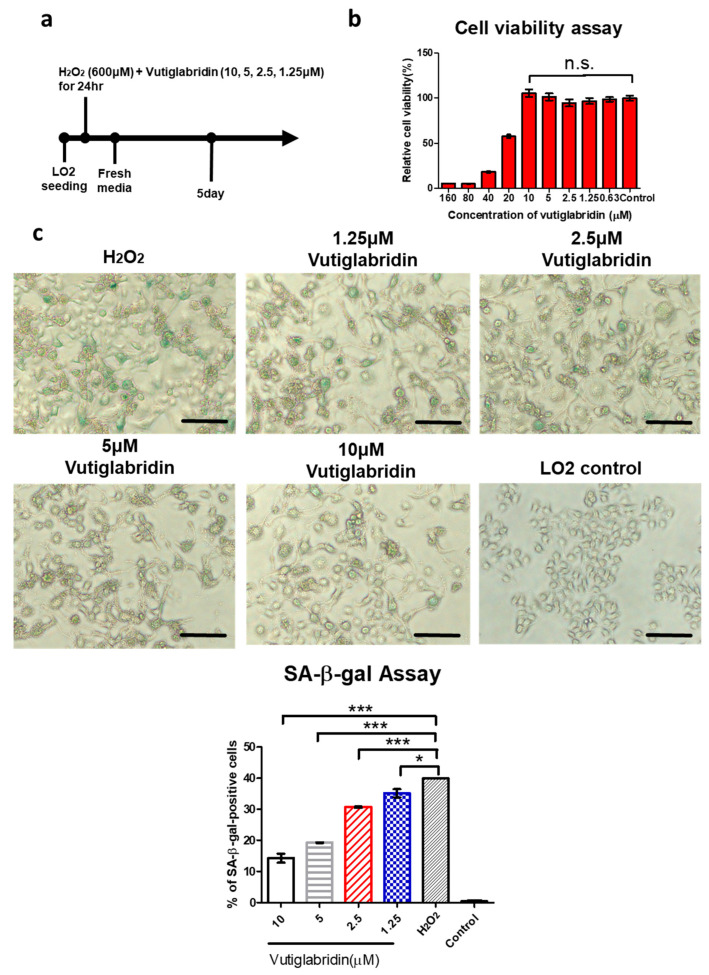

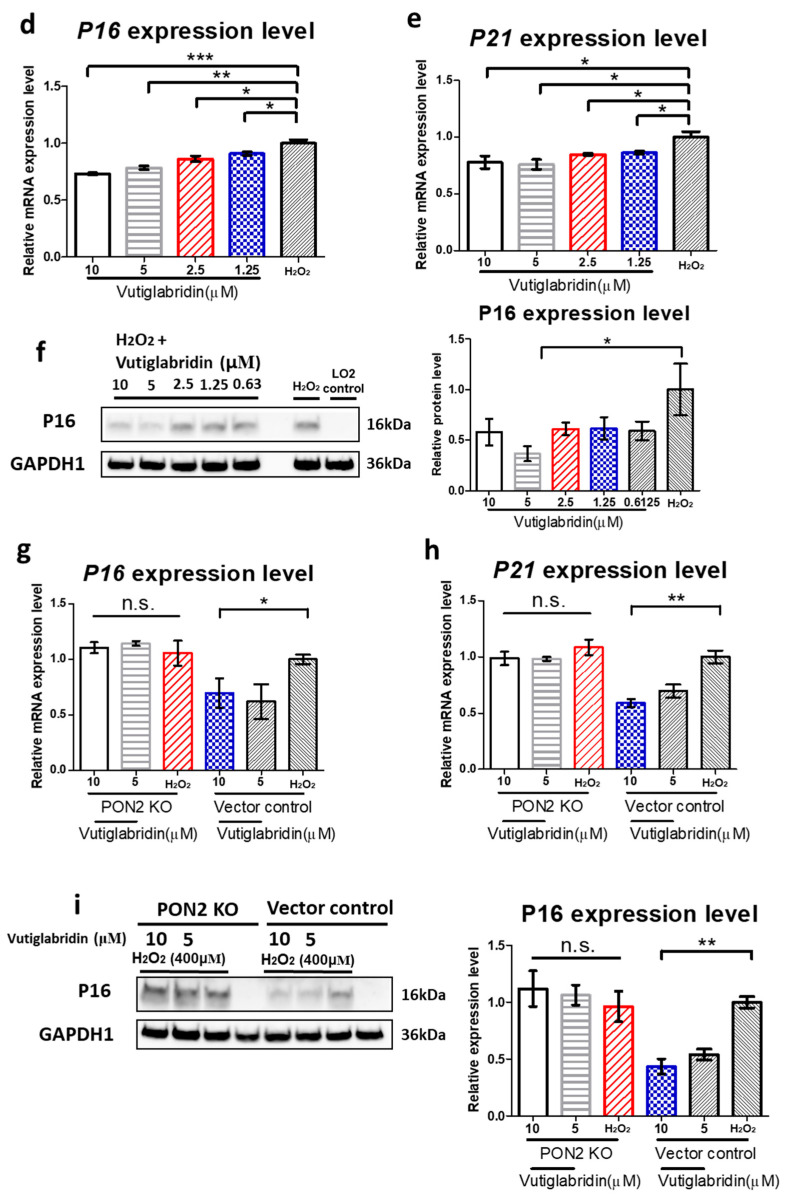
Vutiglabridin mitigates H_2_O_2_-induced senescence in LO2 cells in a PON2-dependent manner. (**a**) Experimental scheme: LO2 cells were treated with 600 μM H_2_O_2_ plus various concentrations of vutiglabridin (10, 5, 2.5, 1.25 μM) for 24 h, followed by incubation in fresh media for 5 days. (**b**) Cell viability assay with various concentrations of vutiglabridin (0.63–160 μM). Relative cell viability was defined as experimental group/control group × 100%. Data are shown as the mean ± SE (n = 6, n.s. no significance). (**c**) Photomicrograph of SA-β-gal staining of LO2s at day 5 after H_2_O_2_ treatment with various concentrations of vutiglabridin. Scale bar = 100 μm. Quantification of SA-β-gal-positive cells was performed from 3 representative fields, with at least 200 total cells analyzed. Results are expressed as the percentage of stained cells. Data are presented as the mean ± SE from three images. (n = 3, * *p* < 0.05, *** *p* < 0.001, by Student’s two-tailed *t*-test). (**d**,**e**) The relative mRNA expression levels of P16 and P21 in LO2 cells treated with H_2_O_2_ and different concentrations of vutiglabridin. Data are shown as the mean ± SE (n = 3, * *p* < 0.05, ** *p* < 0.01, *** *p* < 0.001, by Student’s two-tailed *t*-test). (**f**) Total lysates from LO2 cells treated with H_2_O_2_ and various concentrations of vutiglabridin were subjected to Western blotting using specific antibodies for P16, with GAPDH1 as the loading control. Protein expression levels were quantified relative to H_2_O_2_-group. Data are shown as the mean ± SE (n = 4, * *p* < 0.05, by Student’s two-tailed *t*-test). (**g**,**h**) The relative mRNA expression levels of P16 and P21 in PON2 KO and vector control LO2 cells treated with 400 μM H_2_O_2_ with or without vutiglabridin (10, 5 μM) (* *p* < 0.05, ** *p* < 0.01, n.s. no significance, by Student’s two-tailed *t*-test). (**i**) Western blot analysis of P16 protein expression in PON2 KO and vector control LO2 cells treated with 400 μM H_2_O_2_ with or without vutiglabridin (10, 5 μM). Right panels show quantification of relative protein levels. Data are shown as the mean ± SE (n = 4, ** *p* < 0.01, n.s. no significance, by Student’s two-tailed *t*-test).

To determine the alleviating effect of ROS-induced cellular senescence in LO2 cells, vutiglabridin was treated during hydrogen peroxide treatment (Figure 3a). A 600 μM, 24 h hydrogen peroxide treatment was found to be suitable for ROS-induced cellular senescence, and 5 days of culture with fresh media was sufficient to develop a senescent phenotype in LO2s (Figure 2). Cell viability assays demonstrated that vutiglabridin at concentrations up to 10 μM did not affect LO2 cell viability, confirming the absence of cytotoxicity under these conditions (Figure 3b). LO2s treated with hydrogen peroxide showed fewer senescent phenotypes. Representative images of LO2s exposed to 600 μM hydrogen peroxide with 10, 5, 2.5, 1.25, and 0 μM groups and LO2 control groups are shown in Figure 3c. Following the concentration of vutiglabridin, each group was defined as vutiglabridin 10, 5, 2.5, and 1.25 μM groups and a H_2_O_2_ group. SA-β-gal-positive cells were increased by treatment with hydrogen peroxide in LO2s. When vutiglabridin was treated with hydrogen peroxide at higher concentrations, the proportion of SA-β-gal-positive cells decreased (Figure 3c). An analysis of senescence markers showed that vutiglabridin reduced the expression of P16 and P21 at both the mRNA in a dose-dependent manner. A quantitative RT-PCR revealed that P16 and P21 mRNA levels were significantly lower in vutiglabridin-treated groups compared with the H_2_O_2_ group, where vutiglabridin 1.25 μM groups showed significantly lower mRNA levels than the H_2_O_2_ group, with 0.91- and 0.86-fold lower values (Figure 3d,e, * *p* < 0.05). With a higher concentration of vutiglabridin, P16 and P21 mRNA expression levels decreased. Figure 3d shows fold changes in P16 mRNA levels of 0.78 (vutiglabridin 10 μM group), 0.76 (vutiglabridin 5 μM group), 0.85 (vutiglabridin 2.5 μM group), and 0.86 (vutiglabridin 1.25 μM group) relative to the H_2_O_2_ group, respectively. Figure 3e shows fold changes in P21 mRNA levels of 0.73 (vutiglabridin 10 μM group), 0.78 (vutiglabridin 5 μM group), 0.86 (vutiglabridin 2.5 μM group), and 0.91 (vutiglabridin 1.25 μM group) relative to the H_2_O_2_ group, respectively. A Western blot analysis further confirmed that P16 protein levels were also significantly decreased by vutiglabridin treatment (Figure 3f). Vutiglabridin 1.25 μM groups showed significantly lower P16 protein levels than the H_2_O_2_ group, with 0.59- and 0.74-fold lower values (Figure 3f, * *p* < 0.05). With a higher concentration of vutiglabridin, P16 expression levels decreased. Figure 3f shows fold changes in P16 protein expression levels of 0.58 (vutiglabridin 10 μM group), 0.37 (vutiglabridin 5 μM group), 0.61 (vutiglabridin 2.5 μM group), 0.62 (vutiglabridin 1.25 μM group), and 0.59 (vutiglabridin 0.6125 μM group) relative to the H_2_O_2_ group, respectively. To determine whether the anti-senescent effects of vutiglabridin are dependent on PON2, we compared its efficacy in PON2 KO and vector control LO2 cells. In vector control cells, vutiglabridin significantly reduced H_2_O_2_-induced upregulation of P16 with respect to both the mRNA and protein levels and P21 with respect to the mRNA levels. However, these effects were abolished in PON2 KO cells, indicating that PON2 is required for vutiglabridin-mediated inhibition of senescence marker expression (Figure 3g–i). Collectively, these results demonstrate that vutiglabridin effectively attenuates H_2_O_2_-induced cellular senescence in LO2 cells by reducing P16 and P21 expression and preserving normal cell condition and that these protective effects are dependent on PON2.

To determine whether the anti-senescent effects of vutiglabridin are dependent on PON2, we compared its efficacy in PON2 KO and vector control LO2 cells. In vector control cells, vutiglabridin significantly reduced H_2_O_2_-induced upregulation of P16 and P21 at the mRNA level. Specifically, 10 μM vutiglabridin reduced P16 mRNA levels to 0.70-fold and P21 mRNA levels to 0.59-fold compared with the H_2_O_2_ group (Figure 3h,i, * *p* < 0.05, ** *p* < 0.01). Similarly, 5 μM vutiglabridin reduced P16 and P21 mRNA levels to 0.62- fold and 0.70-fold of the H_2_O_2_ group. A Western blot analysis revealed even more pronounced reductions at the protein level in vector control cells, with 10 μM vutiglabridin decreasing P16 protein to 0.44-fold of the H_2_O_2_ group (Figure 3i, ** *p* < 0.01). Treatment with 5 μM vutiglabridin similarly reduced P16 protein levels to 0.54-fold and 0.34-fold. In contrast, vutiglabridin treatment showed no protective effect in PON2 KO cells. P16 mRNA levels in PON2 KO cells treated with 10 μM and 5 μM vutiglabridin were 1.10-fold and 1.14-fold of the H_2_O_2_ group, showing no reduction and slight increase (Figure 3g, n.s.). Similarly, P21 mRNA levels were unchanged at 0.99-fold and 0.98-fold with 10 μM and 5 μM vutiglabridin, compared with 1.09-fold in the H_2_O_2_ group (Figure 3h, n.s.). At the protein level, P16 expression in PON2 KO cells treated with 10 μM and 5 μM vutiglabridin was 1.12-fold and 1.06-fold of the H_2_O_2_ group (0.96-fold). These results clearly demonstrate that the anti-senescence effects of vutiglabridin are dependent on PON2, as these effects were completely abolished in PON2 KO cells. The data suggests that PON2 is required for vutiglabridin-mediated inhibition of senescence marker expression, establishing PON2 as the critical mediator of vutiglabridin’s protective action against oxidative stress-induced cellular senescence.

### 3.4. Vutiglabridin Preserves Mitochondrial Structure and Network Integrity to Attenuate Oxidative Stress-Induced Senescence in LO2 Cells

**Figure 4:**
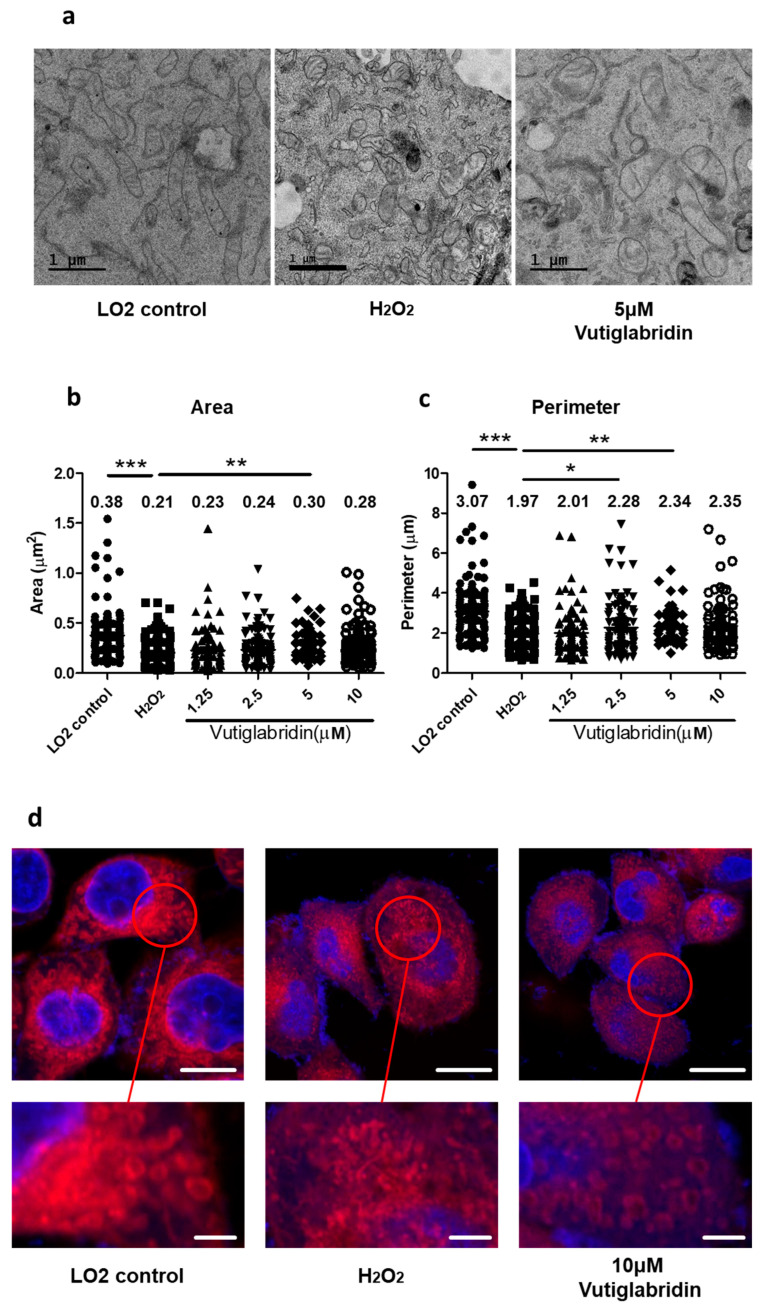
Vutiglabridin preserves mitochondrial morphology and network integrity in H_2_O_2_-treated LO2 cells revealed by TEM and STED microscopy. (**a**) The ultrastructure of mitochondria in electron microscopy. The scale bar is 1 µm. (**b**,**c**) Area or perimeter of ultrastructure of mitochondria in electron microscopy. Each black dot represents different data points. The values at the top of each bar indicate the average value of the corresponding group. The unit in (**b**) is μm^2^ and the unit in (**c**) is μm (n = 60–100; * *p* < 0.05, ** *p* < 0.01, *** *p* < 0.001, by Student’s two-tailed *t*-test). (**d**) Diffraction-unlimited stimulated emission depletion microscopy of mitochondrial networks in LO2 cells. Red: MitoTracker (mitochondria); blue: Hoechst 33342 (nuclei). Views showing the magnified red circle area in the upper panel (lower panels). The scale bars in the upper panels represent 5 μm, whereas those in the lower panels represent 1 μm.

Mitochondrial dysfunction is a key feature of cellular senescence and is reflected by both structural and functional decline, including mitochondrial fragmentation, swelling of the matrix, and disruption of cristae organization [30]. These morphological aberrations are mechanistically linked to senescence through persistent mitochondrial ROS (mtROS) overproduction, which induces DNA damage response pathways and activates p53/p21-mediated growth arrest [31]. To investigate whether vutiglabridin’s anti-senescent effects involve preservation of mitochondrial morphology, we examined mitochondrial ultrastructure using TEM and analyzed mitochondrial networks through diffraction-unlimited STED microscopy. Overall, fragmented cell debris and shortened mitochondria, visibly reduced cristae disruption, mitochondrial swelling, and increased autophagic vesicles were observed in the hydrogen peroxide-treated LO2 groups (Figure 4a). The ImageJ macro tool was used to quantify mitochondrial morphology [32]. Hydrogen peroxide treatment of LO2s significantly decreased the respective mitochondrial area and perimeter (Figure 4b, control group: 0.38 μm^2^, H_2_O_2_ group: 0.21 μm^2^, *** *p* < 0.001) (Figure 4c, control group: 3.07 μm, H_2_O_2_ group: 1.97 μm, *** *p* < 0.001). However, the vutiglabridin-treated LO2 groups showed different characteristics of mitochondria. The area and perimeter of mitochondria in vutiglabridin groups were increased compared with H_2_O_2_ group with respect to their concentration of vutiglabridin. In Figure 4b, the average mitochondrial area was 0.23, 0.24, 0.30, and 0.28 μm^2^ in the vutiglabridin 1.25, 2.5, 5, and 10 μM groups, respectively, compared with 0.21 μm^2^ in the H_2_O_2_ group. In Figure 4b, the average mitochondrial perimeter was 2.01, 2.28, 2.34, and 2.35 μm in the vutiglabridin 1.25, 2.5, 5, and 10 μM groups, respectively, compared with 1.97 μm in the H_2_O_2_ group. Overall, considering the area and perimeter of mitochondria, vutiglabridin reduced ROS damage to cells and mitochondria during hydrogen peroxide treatment and alleviated mitochondrial morphological changes of LO2s. To further evaluate mitochondrial network organization, we employed STED super-resolution microscopy with MitoTracker labeling. Control LO2 cells displayed an interconnected mitochondrial network with an elongated tubular structures. In contrast, H_2_O_2_-treated cells showed severely fragmented mitochondria with a punctate appearance and loss of network connectivity. Co-treatment with 10 μM vutiglabridin substantially prevented this network fragmentation, maintaining mitochondrial connectivity and tubular morphology despite oxidative stress (Figure 4d). These findings demonstrate that vutiglabridin effectively preserves mitochondrial structural integrity during oxidative stress-induced senescence, which may contribute to its protective effects against cellular senescence. The maintenance of proper mitochondrial morphology likely supports organelle function and prevents the metabolic dysregulation typically associated with the senescent phenotype.

## 4. Discussion

This study highlights the therapeutic potential of vutiglabridin in mitigating oxidative stress-induced cellular senescence through PON2. The findings demonstrate that vutiglabridin significantly reduces ROS, attenuates expression of senescence-associated markers (p16 and p21), and preserves mitochondrial morphology and function in a PON2-dependent manner.

ROS are not merely byproducts of mitochondrial metabolism but rather act as central mediators in activating DDR pathways, particularly ATM/ATR and NF-κB. These, in turn, reinforce the SASP and promote further mitochondrial dysfunction. The present study lends support to this paradigm, demonstrating that H_2_O_2_-induced oxidative stress elevates intracellular ROS, upregulates p16/p21 expression, and leads to characteristic mitochondrial alterations in LO2 cells. To confirm further, Western blotting for p21 is required, and this should be performed afterwards. LO2 cells are relatively resilient to oxidative insults due to their expression of enzymes like catalase, glutathione peroxidase, and superoxide dismutase. Therefore, inducing visible oxidative damage or apoptosis typically requires higher H_2_O_2_ concentrations (400–800 μM) compared with more sensitive cell types such as fibroblasts. Another study conducted a similar scheme of experiments using 400 μM H_2_O_2_ in LO2 cells [33]. In our experiment, when comparing 400 μM and 600 μM, there was no significant difference in cell viability, so we selected 600 μM to induce more definitive oxidative stress and proceeded with the experiment.

PON2 has been implicated in a number of biological processes, including cellular antioxidant defense, mitochondrial integrity, and lipid metabolism. The present findings are consistent with earlier reports that PON2 localizes to the inner mitochondrial membrane and regulates coenzyme Q-dependent redox cycling, thereby mitigating mitochondrial ROS generation [24]. In the present study, PON2 KO cells demonstrated an impaired capacity to respond to vutiglabridin, thereby underscoring the critical role of PON2 in vutiglabridin’s mechanism of action.

It is important to note that the administration of vutiglabridin resulted in a reduction of senescence marker expression, exclusively in PON2-intact cells. The absence of effect in PON2 KO cells serves to confirm the specificity of this particular pathway. The capacity of vutiglabridin to maintain mitochondrial area and perimeter, as evidenced by TEM and STED imaging, further underscores the notion that mitochondrial structural integrity constitutes a pivotal element of its protective mechanism. These morphological outcomes are concomitant with functional improvements, such as the restoration of mitochondrial network connectivity and the reduction of autophagic vesicle formation [34].

The findings of this study provide evidence to support a dose-dependent effect of vutiglabridin. It was demonstrated that even low concentrations (1.25 μM) resulted in a measurable decrease in senescence markers, while higher doses elicited more pronounced effects. This graded response further supports the therapeutic potential of vutiglabridin in modulating redox balance and cellular senescence.

Another notable finding is that PON2 deficiency abrogated vutiglabridin’s protective effects upon H_2_O_2_ exposure. This finding indicates that baseline PON2 activity is indispensable for maintaining redox equilibrium. The upregulation of P16 and P21, as evidenced by the observations, in PON2 KO cells suggests that in the absence of this antioxidant safeguard, cells are more susceptible to oxidative insults and are more likely to enter a senescent state. To confirm this more firmly, Western blotting for p21 is necessary and should be performed in future studies.

The preservation of mitochondrial morphology and function is imperative in preventing cellular senescence. The present study utilized super-resolution microscopy to observe the effects of vutiglabridin on mitochondrial networks. The results indicated that vutiglabridin promoted the maintenance of healthy mitochondrial networks, which possibly have led to a reduction in the necessity for compensatory mitophagy. These findings are consistent with emerging views that mitochondrial fragmentation and loss of network integrity are early indicators and drivers of the senescent phenotype. However, further detailed analysis is required to determine whether mitochondrial dysfunction is simply a result of oxidative stress caused by H_2_O_2_ or whether this dysfunction promotes other cellular senescence processes. In addition, further analysis of mitochondrial functional damage through OCR/ECAR is required to provide more specific functional changes in the future.

Taken together, the present study positions PON2 as a pivotal node in the ROS–mitochondrial dysfunction–senescence axis and identifies vutiglabridin as a potent agent with the capacity to restore mitochondrial homeostasis. The therapeutic relevance of targeting PON2 extends beyond hepatocytes, as previous studies have linked its deficiency to pathologies in the retina [18] and cardiovascular system [35]. Meanwhile, it is worth noting that a recently published research paper suggests the possibility that LO2 may have been contaminated with Hela cells rather than hepatocytes [36].

To generalize the conclusions proposed here, experiments using the same scheme should be conducted on different types of cells to determine the extent of vutiglabridin’s efficacy. In addition, future studies should address the long-term effects of vutiglabridin treatment in vivo and assess its potential to delay or reverse tissue-specific aging phenotypes in a PON2-dependent manner. Additionally, elucidating the precise molecular interactions between vutiglabridin and PON2, binding affinity, and downstream signaling cascades including off-target effects has the potential to facilitate the development of more selective agonists.

## 5. Conclusions

The data presented herein provide compelling evidence that vutiglabridin alleviates H_2_O_2_-induced cellular senescence in a PON2-dependent manner, suppressing senescence marker expression and preserving mitochondrial structure and function. These findings presented here suggest that PON2, which is associated with cellular senescence that causes age-related pathologies, is a promising therapeutic target, and that vutiglabridin, which specifically regulates PON2, may alleviate these pathologies by improving cellular senescence and mitochondrial dysfunction.

## Data Availability

The datasets used and analyzed during the current study are available from the corresponding author upon request.

## Abbreviations

The following abbreviations are used in this manuscript:

PON2	Paraoxonase 2
ROS	Reactive oxygen species
TEM	Transmission electron microscopy
STED	Stimulated emission depletion
SA-β-gal	Senescence-associated β-galactosidase
CDKIs	Cyclin-dependent kinase inhibitors
SASP	Senescence-associated secretory phenotype
DDR	DNA damage response
SAMD	Senescence-associated mitochondrial dysfunction
RPE	Retinal pigment epithelial
BMAL1	Basic helix–loop–helix ARNT like 1
KO	Knockout
